# Bryophyte Diversity and Distribution Patterns along Elevation Gradients of the Mount Etna (Sicily), the Highest Active Volcano in Europea

**DOI:** 10.3390/plants12142655

**Published:** 2023-07-15

**Authors:** Marta Puglisi, Saverio Sciandrello

**Affiliations:** Department of Biological, Geological and Environmental Sciences, University of Catania, Via A. Longo 19, 95125 Catania, Italy; s.sciandrello@unict.it

**Keywords:** bryophyte flora, chorotypes, conservation, diversity, elevation gradients, life forms, life strategies, hotspot areas

## Abstract

Mt Etna in Sicily hosts a bryophyte floristic richness of 306 taxa, corresponding to 259 mosses, 43 liverworts, and 4 hornworts. Species richness shows a hump-shaped relationship with the elevation, with a peak at 1200–1700 m a.s.l. Chorotype patterns clearly change along an altitudinal gradient, from the Mediterranean, located at 0–300 m a.s.l., to Arctic-montane and boreo-Arctic montane at 1800–2700 m a.s.l., showing a correlation with the bioclimatic belts identified for the Mt Etna. In regard to the life form pattern, the turf species are the most represented in each elevation gradient, except at 2300–2700 m a.s.l. where the tuft species are prevalent. The life strategy pattern shows the colonists as the prevailing species, featured by an increasing trend up to 2200 m of elevation; above this limit, they are exceeded by the perennial stayers. Furthermore, taking into consideration the red-listed species (at the European and/or Italian level), as well as the species of phytogeographical interest, it was possible to identify the high bryophyte conservation priority areas; these areas are located in thermo-Mediterranean and oro-Mediterranean bioclimatic belts, the latter corresponding to the oldest substrates of the volcano where some of the most interesting bryophyte glacial relicts find refuge.

## 1. Introduction

Bryophytes are the most diverse group of land plants after the flowering plants. They are significant contributors to diversity in many temperate ecosystems and play a crucial role in ecosystem functioning [1]. Bryophytes are poikilohydric and lack roots and, in most cases, an outer waxy cuticle; they take up water and nutrients directly through their surface [2]. Unlike tracheophytes, they are particularly sensitive to the microclimate, mainly moisture, because they lack control over water loss which they compensate for with different strategies, using physiological mechanisms [3] and colony architecture [4] to increase desiccation tolerance. They are more sensitive to environmental changes because of their simple anatomy (absence of roots and an efficient vascular system) and because they absorb water and nutrients from their entire surface; therefore, global warming and climate change have significant impacts on them. One of the effects on bryophytes is the alteration of their distribution patterns [5], with shift to higher elevations or latitudes in search of suitable habitats. As they have a high sensitivity to climatic conditions, widespread distribution, and are found from the tropics to the polar regions, from sea level to above the tree line, they have been advanced as useful organisms to be used in latitudinal and altitudinal studies [6]. In particular, elevation gradients are among the most suitable model templates for testing species diversity distribution–climate relationships, reflecting the adaptive traits or life strategies of species found at different elevations. In mountain areas, the elevation gradient causes changes in moisture, temperature, precipitation, and solar radiation. Thus, the knowledge of richness, abundance, and distribution of species and their assemblage along the altitudinal gradients provide us with data to study the relationships between species diversity and climate change [7,8,9] and improve conservation strategies [10]. The many studies to date have focused on relatively few taxonomic groups, in particular vascular plants, invertebrates, and vertebrates [11]. Research on bryophyte diversity along elevation gradients is mainly focused on large mountains in various parts of the world, including tropical zones, Alps, and central Apennines [8,11,12,13,14,15]., while the Mediterranean islands have received no attention.

Sicily, the largest Mediterranean island, is considered one of the main biodiversity hotspots in the Mediterranean region [16,17,18,19], with about 3250 native and naturalized taxa of vascular plants [20] and 600 bryophytes (117 liverworts, 4 hornworts, and 479 mosses) [21]. Mt Etna, as a geologically recent volcano (Late Quaternary), located in eastern Sicily, is very interesting for the study of plant colonization processes and speciation mechanisms, both of which are favored by its important altitudinal development (highest peak at 3328 m a.s.l.), geographic isolation (i.e., insular mountain system causing a “double insularity”), geo-lithological isolation, and the incessant volcanic activity leading to a continuous creation of new, bare land. This high Mediterranean mountain hosts a set of phytogeographically interesting bryophytes (artic-montane, boreo-Arctic montane, and boreal-montane species) that find on the Mt Etna their southernmost distribution limit.

## 2. Results

**Figure 1:**
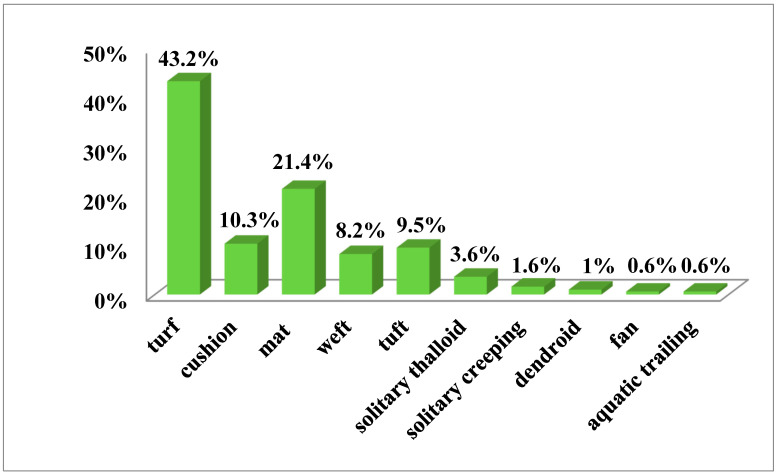
Life form spectrum of the Mt Etna bryophyte flora.

**Figure 2:**
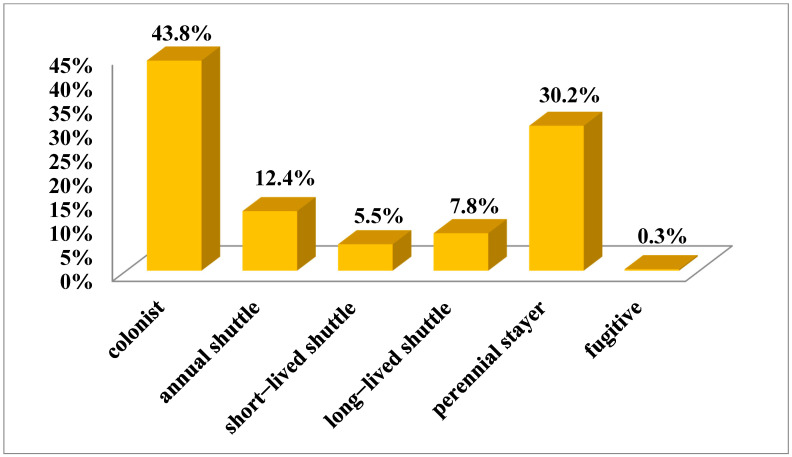
Life strategy spectrum of the Mt Etna bryophyte flora.

Based on the collected data, the bryophyte flora of Mt Etna accounts for 306 taxa (259 mosses, 43 liverworts, and 4 hornworts), together belonging to 56 families. The most represented are Pottiaceae (71 taxa, 23.2%), Brachytheciaceae (28 taxa, 9.2%), Grimmiaceae (26 taxa, 8.5%), Bryaceae (17 taxa, 5.6%), and Mniaceae (15 taxa, 4.9%). The life form spectrum of the bryophyte flora indicates prevalence of turf species (40.7%), followed by mat (21.8%), cushion (11.3%), tuft (10.5%), and weft (8.0%) (Figure 1). The life strategy spectrum shows the higher incidence of colonist (42.2%) and perennial stayer (31.3%) species (Figure 2).

### 2.1. Elevational Gradient of Species Richness

**Figure 3:**
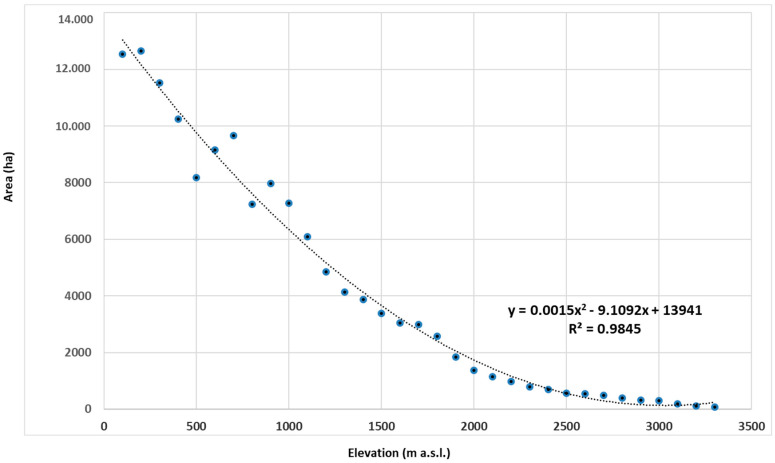
Elevational gradient of each belt on Mt Etna.

**Figure 4:**
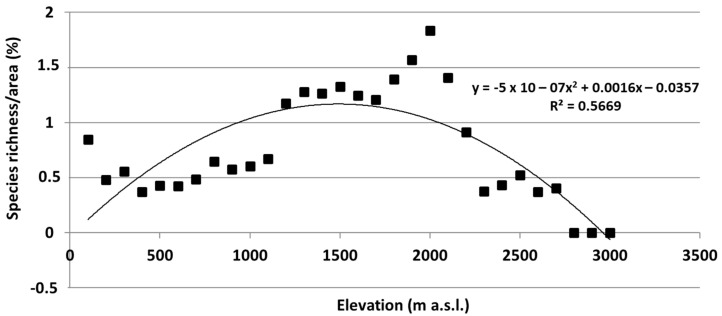
Elevation gradient of Mt Etna bryophyte species richness.

Overall, with increasing elevation, the area for each altitudinal belt declines almost constantly (Figure 3). The bryophyte richness distribution, in relation to the belt area expressed in percentage (Figure 4), shows a parabolic, unimodal (hump-shaped) relationship with the elevation, showing a peak of richness at 1200–1700 m a.s.l.; then, from 1700 to 2700 m a.s.l., a growing reduction in species richness occurs.

### 2.2. Cluster Analysis

**Figure 5:**
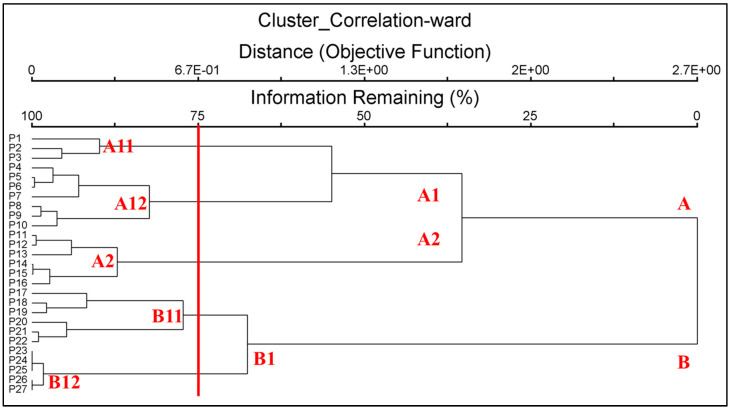
Cluster analysis of the Mt Etna bryophytes in relation to the altitudinal range.

Floristic similarity across the different elevational gradients was examined using hierarchical cluster analysis (Figure 5). All flora species are joined in clusters corresponding to the mountain ranges in which they occur.

The results of the cluster analysis show two main, well separated, and not correlated floristic groups: one with species richness on gradients of low and medium elevation to 1600 a.s.l. (cluster A), and the other at high elevations, to 2700 m (Cluster B). Cluster A is subdivided in two groups of clusters (A1 and A2), the first one comprising the bryophytes of the belts 0–1000 m a.s.l., and the second one the bryophytes of 1000–1600 m a.s.l. In particular, cluster A1 comprises the clusters A11 (species occurring at 0–300 m a.s.l.) and A12 (species occurring at 400-,1000 m a.s.l.). Cluster B includes only the cluster B1, subdivided into B11 (species occurring at 1700–2200 m a.s.l.) and B12 (species occurring at 2300–2700 m a.s.l.). The highest similarity is shown by the species of the cluster B12, represented by a few species, occurring in a few refuge stations.

### 2.3. Life form Distribution Patterns

**Figure 6:**
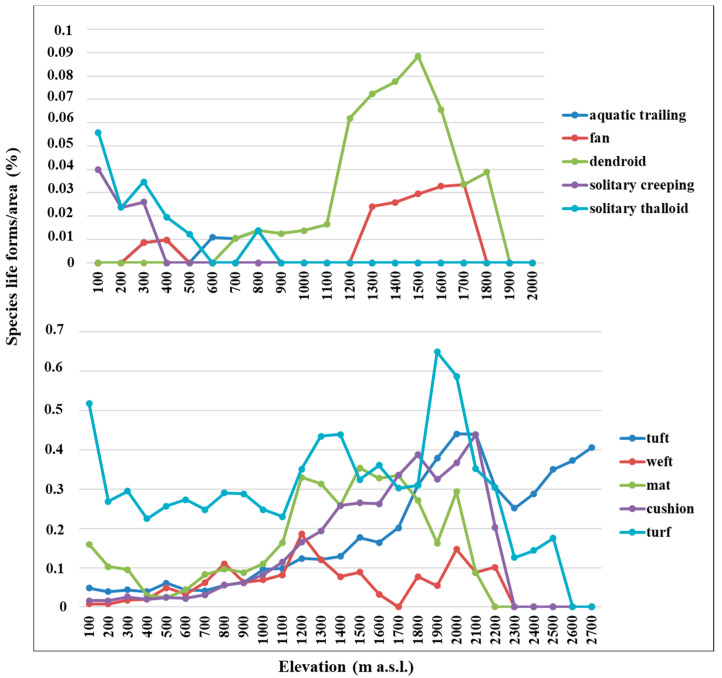
Life forms of the Mt Etna bryophyte flora in relation to elevation.

Life form distribution patterns along the altitudinal gradient of the whole Etnean flora are shown in Figure 6 as percentage of each life form category per altitudinal interval related to the area of each altitudinal belt. Turf species (including turf protonemal and turf scattered) are the dominant life form, accounting for 40.7% of the whole bryoflora; they tend to increase up to 1900 m a.s.l., the latter representing their peak, and then they rapidly decrease up to 2700 m a.s.l. Cushion species, very scarcely present up to 700 m a.s.l., increase up to 2100 m a.s.l. and then abruptly decrease up to 2300 m a.s.l. Mat species show a good presence at 100–300 m a.s.l. and at 1200–2000 m a.s.l., then decrease. Dendroid and fan species, mostly represented on the Mt Etna by epiphytes, mainly occur between 1200 m and 1800 m a.s.l. Tuft species, scarcely occurring at 0–900 m a.s.l., show an increasing trend, and dominate at 2300–2700 m a.s.l. 

Overall, the lower elevations (<500 m) are characterized by an abundance of turf species and exclusive presence of solitary thalloid (especially *Riccia* spp.) and solitary creeping species (*Fossombronia* spp.); moreover, a lot of mat thalloid species occur too. The elevations from 600 m to 1200 m a.s.l. are characterized by a dominance of weft species; turfs and mats are represented with lower percentages. Above 1300 m a.s.l., turf, cushions, and mats are dominating.

### 2.4. Life Strategy Distribution Patterns

**Figure 7:**
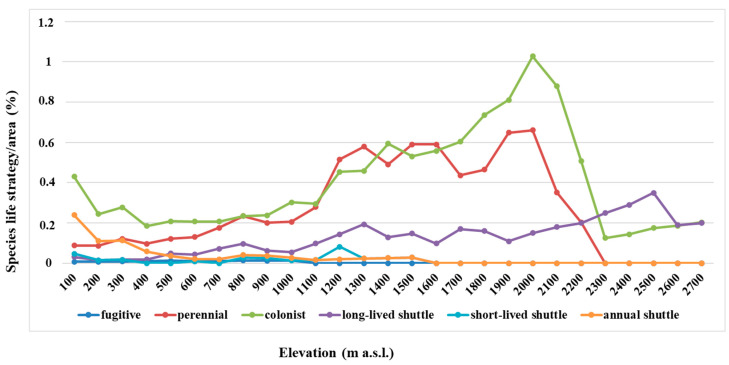
Life strategy of the Mt Etna bryophyte flora in relation to elevation.

Colonist species, amounting to 42.2% of the total, are the dominant life form and tend to increase up to 2000 m a.s.l., and then they decrease (Figure 7). Perennial stayer species, accounting for an average of 31.3%, behave similarly to colonist taxa, also peaking at 2000 m a.s.l.. The long-lived shuttle strategy, accounting for 8.2%, show an increasing trend up to 2400 m a.s.l. Short-lived shuttle species (6.1%) gradually decrease with increasing elevation up to 700 m a.s.l., increasing up to 1200 m a.s.l. and then decreasing again up to 1600 m a.s.l. Annual shuttle species (11.9%), mostly concentrated at 100–300 m a.s.l., decrease at a constant rate up to 1600 m a.s.l. The fugitive category is represented only by the moss species *Funaria hygrometrica* Hedw. occurring at 100–1000 m a.s.l.

Overall, colonist and perennial species are the most representative life strategies in almost all elevation ranges, with colonists prevailing from 1700 m a.s.l. and representing the only life strategy, together with the long-lived shuttle one, from 2300 m to 2700 m a.s.l.. At low elevations, up to 300 m a.s.l., in addition to colonists, the annual shuttle strategy prevails, mostly represented by liverworts and hornworts with a Mediterranean distribution.

### 2.5. Chorotype Distribution Patterns

**Figure 8:**
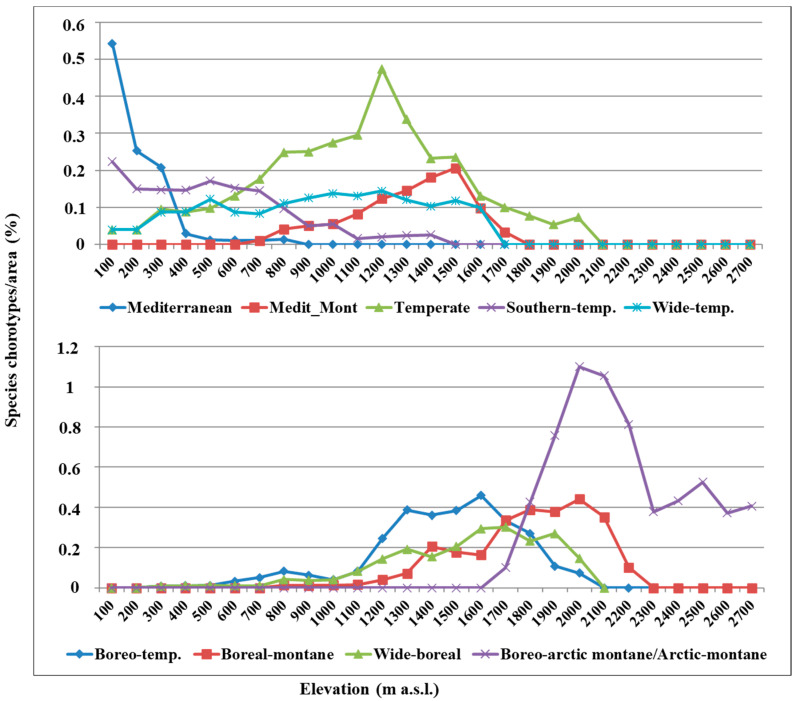
Chorotypes of the Mt Etna bryophyte flora in relation to elevation.

Chorotype distribution patterns along the altitudinal gradient are shown in Figure 8. At low elevations, up to 300 m a.s.l., Mediterranean species distinctly prevail with a peak at 100 m a.s.l., then rapidly decrease up to 800 m a.s.l. The Southern temperate species mostly occur up to 700 m a.s.l., with a peak at 100 m a.s.l., decreasing up to 1400 m a.s.l. Wide temperate species are fairly equally distributed from 300 m a.s.l to 1600 m a.s.l., with low-altitude peaks at 100–200 m a.s.l. and high-altitude peaks at 1000–1200 m a.s.l. Temperate species increase up to 1200 m a.s.l., the latter representing the highest peak, and then rapidly decrease up to 2000 m a.s.l. Mediterranean montane species appear at 700 m a.s.l, with a peak at 1500 m a.s.l., and decrease up to 1700 m a.s.l. Boreo-temperate and wide boreal species are scarcely present at 300–1000 m a.s.l., then increase up to 2000 m a.s.l, with a peak at 1600 m a.s.l. Boreo-temperate, scarcely occurring up to 1100 m a.s.l., increase up to 1600 m and then decrease up to 2000 m a.s.l. The wide boreal species behave similarly to the boreo-temperate taxa, with a peak at 1700 m a.s.l. The boreal montane species, scarcely occurring at 800–1000 m a.s.l., increase up to 2000 m a.s.l. and then rapidly decrease up to 2200 m a.s.l. Finally, boreo-Arctic montane and Arctic-montane occur at 1700–2500 m a.s.l. with a peak at 2000–2100 m a.s.l. 

**Figure 9:**
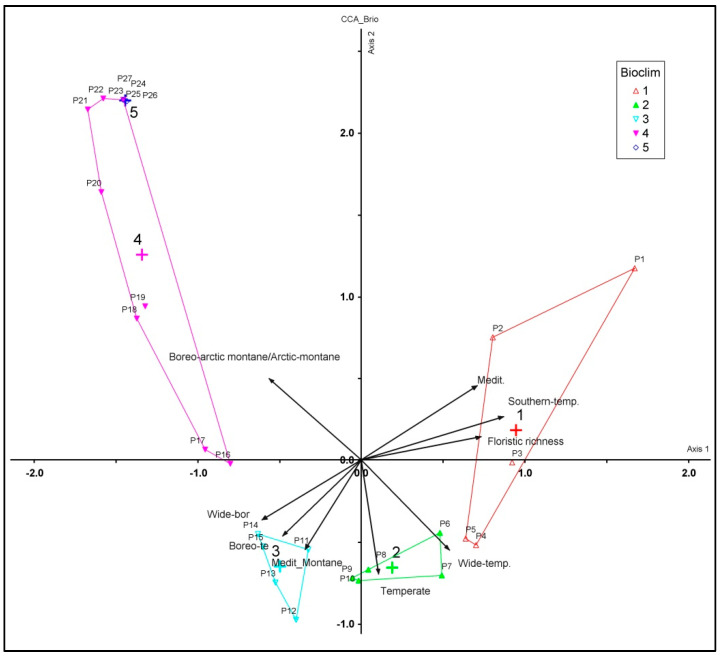
Canonical Correspondence Analysis (CCA). Total variance (inertia) in the species data: 5.75; Eigenvalues: axis 1 = 0.86; axis 2 = 0.73, Variance in species data % of variance explained 14.9 (axis 1), 12.8 (axis 2), Cumulative % explained 14.9 (axis 1) and 27.7 (axis 2).

To investigate the correlation between the chorotypes of the Etnean bryophytes and the bioclimatic belts, a CCA ordination was performed. As shown in Figure 9, the thermo-Mediterranean belt, in the altitudinal gradient 0–500 m a.s.l., hosts Mediterranean and southern temperate species. In the meso-Mediterraneanbioclimatic belt, at 600–1000 m a.s.l., the temperate and wide-temperate species are found, and in the supra-Mediterranean bioclimatic belt, at 1000–1600 m a.s.l., the Mediterranean montane, boreo-temperate, and wide boreal species occur. Finally, in the oro-Mediterranean (1600–2400 m a.s.l.) and crioro-Mediterranean (above 2500 m a.s.l.) bioclimatic belts, only boreo-Arctic montane and Arctic montane species occur.

## 3. Discussion

### 3.1. Degree of Species Richness

Distribution of species richness along elevational gradients, related to the area of each altitudinal belt, shows a hump-shaped relationship with elevation. This pattern recorded on Mt Etna is in accordance with other studies on bryophytes along elevational gradients on several islands, such as La Réunion [12], La Palma (Canary Islands) [13], Pico Island (Azores Islands) [14], and in the continental transects in Colombia [12]. Along the 27 altitudinal intervals of the elevation transect on Mt Etna, many species of bryophytes can be observed. However, the maximum richness was achieved at the middle of the gradient, where the greater concentration of beech woods and deciduous oak woods with *Quercus congesta* C.Presl or *Quercus cerris* L. provide more suitable habitats for bryophyte colonization. In these sites, favorable climatic conditions, mostly related to water availability (precipitation and relative humidity due the presence of woods), are decisive for the effective colonization of bryophytes. The increasingly harsher ecological conditions, with less potential area for growth, toward greater elevations leads to decreasing species richness. Overall, the area available for plant colonization, on a high active volcano such as Mt Etna, is not constant due to the sudden fallout of lava flows or tephra falls that may suddenly cover huge surfaces or due to catastrophic natural events that may reduce the available area because of huge collapses, as it occurred 15,000 years ago on Mt Etna. In addition, such variations may mainly affect areas at high elevations and, subsequently, cause variations in total plant species richness and degree of isolation. Despite this, Mt Etna hosts a rich bryophyte flora corresponding to more than a half of the Sicilian flora [21].

### 3.2. Species of Conservation Interest

Our study revealed important data regarding the distribution pattern and preferential habitats of some rare bryophytes [22] included in the Italian and European Red Book. Overall, on the Mt Etna, 26 species are considered threatened or near threatened at the European and/or Italian level (Table 1), corresponding for 8.5% of the whole Etnean flora. Four species are categorized as Endangered (EN) in Europe, six as Vulnerable (VU), and six as Near Threatened (NT); one additional species is classified as Data Deficient (DD). An additional five species, considered Least Concern (LC) at the European level, are classified as Vulnerable in Italy and other five species as Near Threatened. Although the species under NT and DD classifications are not strictly considered threatened, as the term is applied by the IUCN, these categories indicate that these species deserve special attention; in particular, they can fall under a threat category when more information is acquired (DD), or if their habitats change drastically (NT) [23].

Moreover, a set of phytogeographycally interesting species should be considered too. They are species occurring in Sicily, only on Mt Etna, and rare in Italy. They mostly occur at 1700–2100 m a.s.l., and only one species reaches 2700 m a.s.l. Moreover, for some species, the Mt Etna finding stations represent the only ones confirmed in Italy.

Overall, the areas with high concentrations of species of conservation interest correspond to two different bioclimatic belts: the thermo-Mediterranean (0–400 m a.s.l.) and the supra- and oro-Mediterranean (2400–2700 m a.s.l.). The first belt hosts a group of threatened Mediterranean bryophytes that are very rare in Italy, such as *Anthoceros agrestis* Paton, *Exormotheca pustulosa* Mitt., *Riccia warstorfii* Limpr. *ex* Warnst., *Ephemerum serratum* (Hedw.) Hampe, *Ephemerum crassinervium* (Schwägr.) Hampe subsp. *rutheanum* (Schimp.) Holyoak, and *Funariella curviseta* (Schwägr.) Sérgio, etc.

In correspondence with the supra-Mediterranean and oro-Mediterranean bioclimatic belts, the areas with the highest values of rare species are characterized by mesophilous forests dominated by *Fagus sylvatica,* usually occurring between 1600 m and 2000 m a.s.l., referring to the Directive 92/43/EEC priority Habitat 9210* “Apennine beech forests with Taxus and Ilex” or by pine woodlands dominated by *Pinus nigra* subsp. *calabrica* and *Juniperus hemisphaerica,* between 1400 and 1600 m a.s.l., referred to the priority Habitat 9530* “sub-Mediterranean pine forests with endemic black pines”. Other extremely important habitats for the conservation of bryophytes are natural caves, referred to by the Habitat 8320 as “fields of lava and natural excavations”, and to the orophilous pulvinate shrubby with *Astragalus siculus* Biv. or *Juniperus hemisphaerica* Presl., referred to by the Habitat 4090 as “endemic oro-Mediterranean heaths with gorse”. Moreover, the oro-Mediterranean belt corresponds to one of the most active areas of the volcano for the fallout of volcanic ash and partly for volcanic eruptions, the belt corresponding to the oldest substrates of the volcano. In a remarkable variety of natural rocky habitats, here some of the most interesting species find refuge, such as *Brachytheciastrum collinum* (Schleich. *ex* Müll.Hal.) Ignatov & Huttunen, *Grimmia fuscolutea* Hook., *G. alpestris* (F.Weber & D.Mohr) Schleich., *G. donniana* Sm., *G. torquata* Drumm., *Mielichhoferia elongata* (Hoppe & Hornsch. *ex* Hook.) Hornsch., *M. mielichhoferiana* (Funck) Loeske, and *Schistidium flaccidum* (De Not.) Ochyra. At present, they are the most interesting glacial relicts of the Sicilian bryophyte flora.

### 3.3. Life form Distribution Patterns

Life form is an ecological concept embracing structural characters, the aggregation of individuals, and their relationships with environmental demands [24,25]. They are expressions of morphological adaptations to special ecological niches and reflect habitats, being especially related to moisture conditions [26].

Overall, the bryophyte flora of Mt Etna is characterized by the preponderance of turf species, and lower occurrence of the weft and cushion, although cushion species become important in the high mountain ranges. Along the altitudinal transects of Mt Etna, mat thalloid, solitary creeping, and solitary thalloid are characteristic of lowland areas, wefts, dendroid, and fans are typical of montane habitats, whereas higher elevations are rich in cushion, turf, and mats. Moreover, the occurrence of tuft species is quite high at 1900–2700 m a.s.l., mostly represented by cryophytes of montane and high montane areas.

As a general trend, mat-, weft-, tail-, or fan-forming bryophytes prefer shady and humid sites (e.g., forest habitats), whereas cushion- and turf-forming bryophytes (mostly acrocarpous mosses) increase in sun-exposed, xeric sites underlying drought stress [25]; cushions are particularly effective at storing water and are characteristic of habitats with occasional desiccation [27]. Overall, turf and cushion, suitable for reduction of water loss and protection against strong irradiation (drought stress), represent the more competitive life forms at high elevations, because the species with this life form can survive to unsuitable climatic and edaphic conditions like snow cover, strong insolation, and strong winds as occurring on Mt Etna at high elevations. Many tuft-forming bryophytes are cryophytes of montane and high montane areas.

### 3.4. Life Strategy Distribution Patterns

The life strategy system is based on characters such as life span (avoidance vs. tolerance strategy of the gametophyte), breeding system, main reproductive effort (sexual vs. asexual reproduction), and dispersal strategies [numerous small spores (<25 µm) providing chance dispersal vs. few large spores (>25 µm), indicating decreasing long-range dispersal and achory] [28]. Life strategies reflect the ecological site conditions [25] and can be envisaged as a system of co-evolved adaptive traits [29]. On the Mt Etna, colonist and perennial species prevail in almost all elevational ranges. In particular, colonist species (characterized by a rather short life cycle and a high sexual or asexual reproductive effort) are considered as pioneers colonizing harsh environments, where they usually reach very high percentages, such as rocky surfaces, e.g., [30,31,32,33]. On the Mt Etna, many moss species occur on lava surfaces, showing this typical life strategy. The perennial stayer life strategy, characterized by a long life cycle and low reproductive effort, is frequent in late successional stages or long lasting sites under roughly constant environmental conditions, e.g., [33]; many pleurocarpous mosses follow this life strategy. On the Mt Etna, these species are mostly concentrated at 1200–2000 m a.s.l. in humid forests, such as beech woods. Shuttle species are typical of unstable habitats, often underlying anthropogenic disturbance (annual and short-lived shuttle species). The annual shuttle species can be observed in numerous marchantioids of seasonally dry sites, xeric protosoils, amongst rock boulders and exfoliating knobs, underlying disturbance, e.g., [34]; on the Mt Etna, these species, together with short-lived shuttle taxa, are mostly present at low elevations where anthropogenic influence is higher. Long-lived shuttle species require more stable environments where the end of habitat is predictable; many corticolous bryophytes show this life strategy on the mountain areas of Mt Etna. In agreement with Lloret & González-Mancebo [35], who analyzed elevation patterns of life strategy categories in the bryophytes in the Canary Islands, perennial stayers and long-lived shuttle species become established in the upper localities, while many annual shuttle species and colonists become established in the lowest localities. Colonists also occupy the harsh summit in the highest islands.

### 3.5. Chorotype Distribution Patterns

Overall, at low elevations (0–500 m a.s.l.), the Mediterranean species clearly prevail, followed by the southern temperate taxa, present to a lesser extent; also, a small number of wide temperate and temperate species occur. Instead, at medium elevations the temperate species prevail up to 1200 m a.s.l., together with boreo-temperate species up to 1700 m; in the same altitudinal interval (700–1700 m a.s.l.), the Mediterranean montane species appear. Above 1700 m a.s.l., the boreal montane, boreo-Arctic montane, and Arctic montane species prevail; only the boreo-Arctic montane and Arctic montane reach 2700 m a.s.l., beyond which no bryophytes were found.

### 3.6. Cluster Analysis

Overall, it is possible to identify a correlation between the clusters and the bioclimatic belts of the Mt Etna. In particular, cluster A11 includes species of the thermo-Mediterranean belt, with a clear dominance of Mediterranean mosses and liverworts, as well as occurrence of a group of southern temperate and temperate species. Cluster A12 comprises the species of the meso-Mediterranean belt, where the highest presence of southern temperate, temperate, and wide temperate species is found; here, the Mediterranean species disappear. Clusters A11 and A12 share the occurrence of some southern temperate and temperate species with wider altitude ranges. Cluster A2 includes the species of the supra-Mediterranean belt, characterized by the presence of wide boreal, boreo-temperate, and some boreal montane species, nearly absent in the lower altitudinal belts; moreover, a group of wide temperate and temperate species are also present. Occurrence of these last species render cluster A2 somewhat similar to the previous A12 and A11. Cluster B11 includes species of the oro-Mediterranean belt, where the Arctic montane and boreo-Arctic montane species are located, together with boreal montane and wide boreal species; only some boreo-temperate species are still found, while the wide temperate and temperate ones have vanished. Cluster B12 comprises species of the cryoro-Mediterranean belt; these are only a few boreo-Arctic montane species that reach the record altitude of 2700 m a.s.l. The presence of these species approaches the last two clusters (B11 and B12).

## 4. Material and Methods

### 4.1. Study Area

Mount Etna is a large stratovolcano of basaltic nature. Its height varies over time due to its eruptions which cause it to rise or fall. It is a polygenic basaltic volcano covering a surface of 1178 km^2^ from the sea level, along the eastern coast of Sicily; it reaches an altitude of 3328 m a.s.l., being the highest active volcano in Europe and one of the world’s most active volcanoes. At the same time, it represents the highest mountain in Sicily and the highest mountain in Italy south of the Alps. It is characterized by an almost continuous eruptive activity from its summit craters and fairly frequent lava flows from lateral fissures. Its origin is quite recent (late Quaternary), having formed 500 ka ago along a diverging zone in the framework of the Africa–Europe plate convergence [36], with eruptions occurring beneath the sea off the ancient coastline of Sicily. Four main eruptive phases can be recognized, comprising different volcanic successions in which the eruptions occurred with a similar eruptive style in the same geodynamic setting [37,38]. During each phase, several volcanic centers formed, and remnants of these are still recognizable in the field [38]. In particular, after an earlier phase of scattered and discontinuous volcanic activity occurring about 500 ka and 330 ka ago, the volcanism in the Mt Etna region was concentrated along the Ionian Sea coast between 220 and 121 ka ago [38,39]. According to [40], about 80% of the volcanic products were erupted only in the past 110 ka due to the stabilization of the magma source and, 15,000 years ago, the younger volcanics mantled most of the previous edifice (88% of the area) with a widespread cover of lava flow fields and pyroclastic deposits [41]. This, joined to a general process of tectonic uplifting, sometimes broken by the subsidence related to flank sliding of this volcanic edifice, contributes to our understanding of the present structure of the Etna volcano but also to the highlighting of relevant constraints and evolutionary chances for the plants colonizing this mountain in which soils are rejuvenated quite frequently, especially at higher elevations, not only with lava flows, but above all with the fall of volcanic ash and tephra.

As far as the climate is concerned, according to the bioclimatic classification proposed by Rivas-Martinez [42] and Rivas-Martinez et al. [43], Mt Etna is characterized by a Mediterranean pluviseasonal oceanic bioclimate, very diversified in relation to the altitude and exposure. Thermotypes range from the low thermo-Mediterranean to the lower cryo-Mediterranean, while ombrotypes range from the semiarid to the upper hyperhumid [44]. In Sicily, the lower cryo-Mediterranean and upper oro-Mediterranean belts exclusively occur on Mt Etna.

### 4.2. Floristic Data Sources

This research is based on a complete checklist of the Etnean bryophyte flora resulting from literature data, integrated with several field observations conducted in the last three decades all around the volcano by the first author of the paper. Currently, an updated published bryophyte flora of Mt Etna does not exist and, therefore, it was necessary to draw up a preliminary control list and provide accurate distribution data in order to be able to establish the altitudinal range for each taxon. All reports regarding Mt Etna were considered. The first important contribution was from Strobl [45], with the reports of about fifty bryophytes. Some papers concern some restricted Etna territories [46,47,48,49,50]. Other sporadic information can be found in larger studies on the bryoflora of Sicily and Italy [51,52,53,54,55,56,57,58,59,60,61,62,63,64,65,66,67,68,69,70,71,72,73,74]. Finally, a number of more recent published floristic and phytosociological contributions were considered [33,34,75,76,77,78,79,80,81,82,83,84]. More than 700 phytosociological published relevés were examined. As far as the floristic reports and phytosociological relevés are concerned, without any explicit altitudinal indication but only the locality (i.e., toponym), elevation was determined by consulting topographic maps at 1:25,000 or 1:50,000 provided by the I.G.M. (Istituto Geografico Militare_Military Geographic Institute). Combining these data, also checking the taxonomic and nomenclatural correspondences, it was possible to establish a reliable distribution and altitudinal range for each species.

Mt Etna (0–3357 m), according to Trigas, Panitsa, and Tsiftasis [85] and Sciandrello et al. [86], was divided into 33 belts 100 m wide and the bryophyte diversity of each altitudinal belt was calculated as the total number of species per interval. All species were considered to have a continuous distribution between their minimum and maximum elevation limits. The area of each altitudinal belt was calculated using digital elevation models in ArcGIS 10.3 (3D Analyst).

For each species chorotypes, ecological indices, life forms, and life strategy were considered. The chorotypes follow Hill et al. [87], according to the classification of Hill and Preston [88], where the species are assigned to biome categories corresponding to the biomes in which they are found. The classification is as follows: Arctic-montane (with main distribution in tundra or above tree-line in temperate mountains), boreo-Arctic montane (in tundra and coniferous forest zones), wide-boreal (from temperate zone to tundra), boreal-montane (main distribution in coniferous forest zone), boreo-temperate (in conifer and broadleaf zones), wide temperate (from Mediterranean region to coniferous forest zone), temperate (in broadleaf forest zone), southern-temperate (in the Mediterranean region and broadleaf forest zones), Mediterranean (in the Mediterranean region).

Life forms were defined according to Hill et al. [87], and life strategies according to Dierβen [89]. The Ellenberg indicator values (moisture, light, soil acidity) were obtained from Hill et al. [87].

Simple scatter plots were used to show the life forms, life strategies, and corotypes along the elevational gradient of Mt Etna. Simple regression analyses were used to correlate total bryophyte flora to log-area of each elevational interval, both as number of species for each altitudinal interval and as distribution of the floristic richness in relation to the real surface of each belt expressed as a percentage. Regression analyses and generalized linear models were performed using the statistical package Past Version 2.17. A multivariate analysis (Linkage method: Ward’s, Distance measure: Correlation) and ordination (CCA) were used to establish spatial patterns about the bioclimatic belts and corotypes. Cluster analysis and ordination of the dataset were performed using PC-ORD 6 software [90].

In order to identify areas with high bryophyte conservation priority, occurrence on the Mt Etna of species considered threatened or near threatened at the European and/or Italian level [23,91], as well the occurrence of rare and very rare species (in Italy and/or in Sicily, or occurring in Sicily only on the Mt Etna) was considered.

The nomenclature of the species and taxonomy follow Hodgetts et al. [92].

## 5. Conclusions

Species distribution models are widely utilized in conservation biogeography due to their ability to estimate potential range shifts for species and communities in response to climate change. Moreover, they have been successfully used to study the distribution of bryophytes in a variety of ecosystems, including identifying areas of high conservation value [93,94]. Consequently, they represent a valuable tool for informing and guiding conservation management planning.

Our contribution wanted to go one step further by using quantitative data on the spatial distribution of the bryophyte species along the altitudinal gradient and highlight areas with a high concentration of species of conservation interest. In particular, the peculiar hump-shaped pattern of floristic richness at medium elevations (1200–1700 m a.s.l.) was highlighted corresponding to the greater concentration of beech and deciduous oak woods that provide more suitable habitats for bryophyte establishment; here, the boreo-temperate species showing turf and mat life forms and colonist and perennial stayer life strategies are prevalent.

The results of the analysis on the species of conservation interest (red-listed and/or phytogeographically interesting) emphasize the fact that the high plant conservation priority areas are located in the oro-Mediterranean (1800–2400 ms.l.m.) bioclimatic belt, where the oldest substrates of the volcano (110–15 ka), which functioned as refuge areas for high-latitude species (Arctic montane, boreo-Arctic montane, and boreal montane), is found; moreover, it should be noted that in the thermo-Mediterranean belt, some red-listed liverworts are found and, therefore, this should also be taken into consideration for conservation actions. The worthy bryophyte diversity of the Mt Etna discussed in this paper confirmed the floristic value of this volcano, as already highlighted by Sciandrello et al. [85] for the vascular flora.

## Figures and Tables

**Table 1:** Bryophytes of conservation interest on the Mt Etna. * Priority Habitat.

*Taxon*	Red-List Europe	Red-List Italy	Phytogeographical Interest	Distribution on the Etna		
				Sites	Altitude Range (m a.s.l.)	Habitat (Directive 92/43/EEC)
*Amphidium mougeotii* (Schimp.) Schimp.	LC	LC	Occurring in Sicily only on the Etna.	Grotta dei Faggi; Grotta delle Palombe; Mts Silvestri; Mts Calcarazzi; Mt Baracca; Mt Pomiciaro; Grotta dei Lamponi; Grotta dei Ladri; Grotta di Casa del Vescovo; Grotta di Cassone; Grotta del Coniglio; Rifugio Citelli; Mt Palestra; Contrada Germaniera; Grotta Intraleo; Grotta Corruccio.	1400–2100	8320, 4090
*Anthoceros agrestis* Paton	NT	NT	Rare in Italy.	Aci Castello; Piano delle Immacolatelle; Catani; Capo Mulini; Grotta Immacolatella IV.	0–300	8320
*Aschisma carniolicum* (F.Weber & D.Mohr) Lindb.	EN	VU	Rare in Italy; occurring in Sicily only on the Etna.	Capo Mulini.	0–100	-
*Aulacomnium androgynum* (Hedw.) Schwägr.	LC	NE	Rare in Italy; occurring in Sicily only on the Etna.	Contrada Giarrita; M. Rinatu.	1000–1700	9210 *
*Brachytheciastrum collinum* (Schleich. ex Müll.Hal.) Ignatov & Huttunen	LC	VU	Rare in Italy; occurring in Sicily only on the Etna.	Mt Palestra; Mt Maletto; Grotta dei Lamponi; Contrada Germaniera; Grotta del Gelo.	1700–2200	8320, 9530 *
*Callicladium imponens* (Hedw.) Hedenäs, Schlesak & D.Quandt	NT	NE	Rare in Italy; occurring in Sicily only on the Etna.	Valle S. Giacomo.	700–800	8320
*Clevea spathysii* (Lindenb.) Müll.Frib.	NT	DD	Very rare in Italy.	Acireale; Aci Trezza.	0–100	-
*Coscinodon cribrosus* (Hedw.) Spruce	LC	LC	Occurring in Sicily only on the Etna.	Grotta Cassone; Mts Silvestri; Mt Palestra; Rifugio Citelli; Mt Baracca; I Dammusi; Valle del Tripodo; Casa del Vescovo; Etna botanical garden "Nuova Gussonea"; Mt Baracca; Mt Pomiciaro; rifugio di M.te Maletto; Grotta dei Tre Livelli; Contrada Giarrita; Grotta di Casa del Vescovo; Mt Arcimis; a nord di Rifugio Sapienza.	1400–2100	8320, 4090, 9210 *, 9530 *
*Cynodontium bruntonii* (Sm.) Bruch & Schimp.	LC	NE	Occurring in Sicily only on the Etna.	Mt Spagnolo; I Dammusi.	1000–1600	9210
*Entosthodon muhlenbergii* (Turner) Fife	NT	NT	Rare in Italy.	Valle S. Giacomo; Mt Minardo.	700–900	9210, 9340
*Ephemerum serratum* (Hedw.) Hampe	LC	VU	Rare in Italy; occurring in Sicily only on the Etna.	Piano delle Immacolatelle; Grotta Immacolatella IV.	300–400	8320
*Exormotheca pustulosa* Mitt.	NT	CR	The Etna locality is the only one confirmed in Italy.	Aci Castello.	0–100	-
*Fossombronia caespitiformis* (Raddi) De Not. ex Rabenh. subsp. *multispira* (Schiffn.) J.R.Bray & Cargill	LC	NT	Rare in Italy.	S. Maria La Stella; Capo Mulini.	0–100	3170 *
*Fossombronia wondraczekii* (Corda) Dumort. ex Lindb.	LC	NT	Rare in Italy.	Aci Castello; Grotta Cantarella.	0–300	8320
*Funariella curviseta* (Schwägr.) Sérgio	VU	VU?	Rare in Italy.	Aci Castello; Acireale	0–100	-
*Grimmia alpestris* (F.Weber & D.Mohr) Schleich.	LC	NT	Rare in Italy; occurring in Sicily only on the Etna.	Etna botanical garden "Nuova Gussonea".	2100–2200	9530 *
*Grimmia crinita* Brid.	VU	VU	Rare in Italy.	Etna botanical garden "Nuova Gussonea".		9530 *
*Grimmia decipiens* (Schultz) Lindb.	LC	NE	Rare in Italy.	Grotta Forcato; Grotta delle Palombe; Ingresso Demanio forestale; Mt Spagnolo; Contrada Tabbutazzo; Contrada Timpazza; Mt Intraleo; Mt Minardo; Fornazzo; Mt Maletto; Mt Guardiola.	1300–1500	8320, 9210 *, 9340
*Grimmia donniana* Sm.	LC	NE	Rare in Italy; occurring in Sicily only on the Etna.	Mt Spagnolo; I Dammusi; Etna botanical garden "Nuova Gussonea"; Mt Minardo; Ingresso Demanio forestale; Mt Maletto; Mt Pomiciaro; strada forestale tra Mt Maletto e Mt Spagnolo.	1800–2200	8320, 9210, 9530
*Grimmia fuscolutea* Hook.	VU	EN	Very rare in Italy; occurring in Sicily only on the Etna.	Mts Silvestri.	2000–2200	8320, 4090
*Grimmia elatior* Bruch ex Bals.-Criv. & De Not.	LC	NE	Rare in Italy; occurring in Sicily only on the Etna.	Contrada Tabbutazzo; Ingresso Demanio forestale; Mt Minardo; Mts Silvestri.	1700–1900	8320, 4090
*Grimmia torquata* Drumm.	LC	VU	Rare in Italy; occurring in Sicily only on the Etna.	Grotta delle Palombe.	1800–1900	8320
*Hydrogonium bolleanum* (Müll.Hal.) A.Jaeger	DD	VU	Rare in Italy.	Fiume Fiumefreddo.	0–100	3280
*Hymenoloma crispulum* (Hedw.) Ochyra	LC	LC	Occurring in Sicily only on the Etna.	I Dammusi.	1900–2100	9210 *
*Isopterygiopsis pulchella* (Hedw.) Z.Iwats.	LC	LC	Occurring in Sicily only on the Etna.	Grotta delle Palombe.	1700–2000	8320
*Mielichhoferia elongata* (Hoppe & Hornsch. ex Hook.) Hornsch.	VU	VU	Rare in Italy; occurring in Sicily only on the Etna.	Mts Silvestri.	1900–2000	4090
*Mielichhoferia mielichhoferiana* (Funck) Loeske	NT	VU	Rare in Italy; occurring in Sicily only on the Etna.	Mt Frumento; Mts Silvestri.	1900–2100	4090
*Physcomitrium eurystomum* Sendtn. subsp. *eurystomum*	VU	CR	The only recent report for Italy.	Botanical garden of Catania.	0–100	-
*Pohlia proligera* (Kindb.) Lindb. ex Broth.	LC	NT	Rare in Italy; occurring in Sicily only on the Etna.	Grotta dei Lamponi; Mts Silvestri.	1800–1900	8320, 4090
*Pohlia wahlenbergii* (F.Weber & D.Mohr)A.L.Andrews *var. calcarea* (Warnst.) E.F.Warb.	LC	NT	Rare in Italy.	Aci Castello.	0–100	-
*Ptychostomum cernuum* (Hedw.) Hornsch.	EN	EN	The Etna locality is the only one confirmed in Italy.	Grotta del Santo.	1200–1300	8320
*Pyramidula tetragona* (Brid.) Brid.	EN	NE	Very rare in Italy; occurring in Sicily only on the Etna.	Aci Castello.	0–100	-
*Rhabdoweisia fugax* (Hedw.) Bruch & Schimp.	LC	NE	Rare in Italy.	Grotta delle Palombe.	1700–1800	8320
*Schistidium flaccidum* (De Not.) Ochyra	VU	NT	Rare in Italy; occurring in Sicily only on the Etna.	Mt Minardo; Ingresso Demanio forestale; Casa del Vescovo.	1700–1800	9340, 9530 *, 8320
*Timmia bavarica* Hessl.	LC	LC	Rare in Italy.	Grotta dei Lamponi.	1700–1800	8320
*Tortula bolanderi* (Lesq. & James) M.Howe	EN	EN	Very rare in Italy.	Grotta Forcato.	600–700	8320
*Tortula hoppeana* (Schultz) Ochyra	LC	VU	Rare in Italy.	Mts Silvestri; nord di Rifugio Sapienza.	2000–2500	4090

## Data Availability

Not applicable.
